# East London’s Homeless: a retrospective review of an eye clinic for homeless people

**DOI:** 10.1186/s12913-016-1295-8

**Published:** 2016-02-16

**Authors:** Penny J. D’Ath, Laura J. Keywood, Elaine C. Styles, Clare M. Wilson

**Affiliations:** Division of Optometry and Visual Science, School of Health Sciences, City University London, Northampton Square, London, EC1V 0HB UK; Division of Optometry and Visual Science, School of Health Sciences, City University London, Northampton Square, London, EC1V 0HB UK; Vision Care for Homeless People, c/o Crisis Skylight, 66 Commercial Street, London, E1 6LT UK; Department of Ophthalmology, Chelsea and Westminster Hospital NHS Foundation Trust, 369 Fulham Road, London, SW10 9NH UK

**Keywords:** Optometry, Eye care, Homeless people, Ophthalmology

## Abstract

**Background:**

There is very little published work on the visual needs of homeless people. This paper is the first study to investigate the visual needs of homeless people in the UK. Although similar work has been done in other countries, this study is unique because the United Kingdom is the only country with a National Health Service which provides free healthcare at the point of access. This study analysed the refractive status of the sample used, determined the demographics of homeless people seeking eye care and established if there is a need for community eye health with access to free spectacle correction in East London.

**Methods:**

This retrospective case study analysed the clinical records of 1,141 homeless people using the Vision Care for Homeless People services at one of their clinics in East London. All eye examinations were carried out by qualified optometrists and, where appropriate, spectacles were dispensed to patients. Data captured included age, gender, ethnicity and refractive error. Results were analysed using two-sample t-tests with Excel and Minitab.

**Results:**

Demographics of age, gender and ethnicity are described. Spherical equivalents (SE) were calculated from prescription data available for 841 clinic users. Emmetropia was defined as SE–0.50DS to +1DS, myopia as SE < −0.50DS, and hyperopia as SE > +1DS.

The majority of clinic users were male (79.2 %, *n* = 923). Approximately 80 % (*n* = 583) of clinic users were white, 10 % (*n* = 72) were ‘black’, 4 % (*n* = 29) ‘Asian’ and the remaining 5.6 % (*n* = 40) were of ‘mixed ethnicity’ and ‘other’ groups. The mean age of females attending the clinic was significantly lower than that of males (45.9 years, SD = 13.8 vs’ 48.4 years, SD = 11.8) when analysed using a two-sample *t*-test (t (317) = 2.44, *p* = 0.02). One third of service users were aged between 50–59 years. Myopia and hyperopia prevalence rates were 37.0 % and 21.0 % respectively. A total of 34.8 % of homeless people were found to have uncorrected refractive error, and required spectacle correction.

**Conclusions:**

This study has identified a high proportion of uncorrected refractive error in this sample and therefore a need for regular eye examinations and provision of refractive correction for homeless people.

## Background

Homelessness has been described as a “shameful national problem” [1]. It is estimated that there are approximately 310,000 to 380,000 single homeless people in England [2]; the majority of whom are male [3]. There are also a significant number of homeless families including an estimated 116,000 homeless children [3]. These numbers are on the rise. In 2014, the number of people sleeping on the streets in London increased by 75 % over a four year period [4].

Whilst some homeless people resort to sleeping rough, a majority of homeless people live in hostels, squats, bed and breakfast accommodation and in insecure conditions with friends or family [5, 6]. Due to their status, the overall health of homeless people tends to deteriorate due to the difficulties they face in accessing regular health care [3, 6, 7]. Homeless people are more likely to suffer from mental health disorders [3, 8, 9], alcohol and substance abuse [8, 10] as well as less commonly encountered medical conditions such as HIV, hepatitis, diabetes [11], tuberculosis [12], peripheral vascular disease and skin disorders [1]. Homeless people also tend to have more eye problems than the general population. Previous studies have found that homeless people have higher rates of cataract, glaucoma and binocular vision problems [13] and are more likely to have uncorrected refractive error [11, 14]. Mortality rates are higher with an average life span of 42 years [3]. Homeless males are 8.3 times more likely to die than 18–24 year olds in the general population [15]. Despite these figures, homeless people are far less likely to receive medical interventions [6, 9] for reasons including being unable to provide a permanent address [16].

It is not clear how many homeless people have access to eye care as there is little information about the visual needs and visual problems encountered in this population. The studies aforementioned were carried out in North America where health care provisions and policies are different to those in the United Kingdom. As vision plays a significant role in quality of life [13], it is important to investigate the visual needs of this already vulnerable population.

Homelessness is a complex issue and varies in degree of severity. Beyond the traditional image of someone with “no fixed abode”, which relates solely to rough sleepers and street homeless, an individual can be classified as homeless if they live in a hostel, B&B or even sofa surfing (on friends and family members’ couches) [5, 6]. For the purposes of this paper, the criterion used to classify a person as homeless was that the person must not have a fixed stable home.

This paper is the first study of the visual status of homeless people based in the UK.

### Aims

The aims of the study were as follows:

- To analyse the refractive status of the sample used;
- Determine the demographics of homeless people seeking eye care in East London;
- Establish if there is a need for community eye health with access to free spectacle correction in East London.

## Methods

### Study design

This retrospective case study examined the clinical records of homeless people using the Vision Care for Homeless People (VCHP) services at one of their clinics in East London. The VCHP mission statement is “homeless and other vulnerable people” [17]. This could also include people who used to be homeless but now are in a flat, but their income is very low and circumstances are vulnerable. It is important to point out that of those homeless people who have been rehoused, a high number of these have lost their dwelling due to being unable to cope with these changes in circumstance. The charity helps these people too. It is, therefore, possible that a small minority of those using the charity have since been rehoused and are settled in their new accommodation but are still using the charity for their eye care. This issue is consistent with other research into homeless people in that any person (homeless or otherwise) may, for example, attend a soup kitchen or clinic intended for homeless users only regardless of whether they would fit into the homeless categories.

This charity offers full eye examinations but does not conduct eye screening events although it does provide education and advice. All eye examinations were carried out by qualified optometrists and where appropriate, spectacles were dispensed to patients. Details of 1,141 electronic records of eye examinations performed between 2003 and 2012 were transferred onto a new computer database, Optix, for analysis.

In order to make an estimation of the number of people who require spectacles, this study used previously determined figures that an uncorrected refractive error of < = − 1DS and > = + 5DS would result in vision of approximately < =6/18 [18, 19].

In keeping with the Declaration of Helsinki (2000), ethical approval was obtained from the City University London Research and Ethical Committee.

### Data analysis

Data captured included age, gender, date of visit and refractive error. Other data was not included as it was not on the database. Results were analysed using Excel and Minitab.

## Results

A total of 1,141 records were analysed for demographic statistics and refractive error.

### Age

The sample comprised of 903 males (79.2 %) and 238 females (20.8 %).

Age details were recorded for 1,112 (97.5 %) of the sample (Table 1). The mean age was 47.9 years (SD = 12.3; range: 22–87 years). The mean age of females attending the clinic was significantly lower than that of males (45.9 years, SD = 13.8 vs’ 48.4 years, SD = 11.8) when analysed using a two-sample *t*-test (t (317) = 2.44, *p* = 0.02.

**Table 1:** Ages of VCHP patients

*Age range*	*M*	*F*	*Total*
	*N* = *884* (*79.5*)	*N* = *228* (*20.5* %)	*n* = *1*,*112* (%)
Age range 19–29	50 (5.7)	29 (12.7)	79 (7.1)
Age range 30–39	154 (17.4)	61 (26.8)	215 (19.3)
Age range 40–49	248 (28.0)	47 (20.6)	295 (26.6)
Age range 50–59	289 (32.7)	52 (22.8)	341 (30.7)
Age range 60–69	109 (12.3)	27 (11.8)	136 (12.2)
Age range 70–79	27 (3.1)	9 (4.0)	36 (3.2)
Age range 80–89	7 (0.8)	3 (1.3)	10 (0.9)
Total	884	228	1,112 (100.0)

The most frequent age group attending were aged between 50 and 59 years old (30.7 %, 341/1,112), closely followed by the 40–49 year olds (26.6 %, 295/1,112) of clinic users. More than 75 % of patients fell into the 30–59 years old bracket (851/1,112). VCHP rarely see people below 18 years of age in their clinics.

### Ethnicity

Ethnicity was recorded in 63 % (724/1,141) of patients (Table 2). The records included are from the period 2003–2012 which may account for some of the missing data. Missing data is common in large data sets with Talbert et al. (2013) reporting that “up to 36 % of eligible cases” have missing data [20]. Over four fifths (583/724) of the clinic users were ‘white’, 10 % (72/724) were ‘black’, 4 % (29/724) ‘Asian’ and the remaining 5.6 % (40/724) were of ‘mixed ethnicity’ and ‘other’ groups. The ‘white other’ category includes people originating from countries apart from the UK or Ireland such as Europe.

**Table 2:** Ethnicity of VCHP patients

Ethnicity	*n* = 724	Total (%)
White Other	355	49.0
White British	206	28.5
Black/British-African	45	6.2
Black/British-Caribbean	24	3.3
White Irish	22	3.0
Asian/British-Other	13	1.8
Mixed White & Black African	10	1.4
Asian/British-Indian	10	1.4
Other	10	1.4
Mixed Others	8	1.1
Chinese	6	0.8
Mixed White & Black Caribbean	4	0.6
Asian/British-Pakistani	3	0.4
Asian/British-Bangladeshi	3	0.4
Black/British-Other	3	0.4
Mixed White & Asian	2	0.3
Total	724	100.0

### Number of eye examinations

**Fig. 1:**
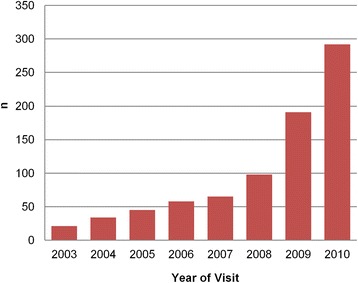
Number of eye examinations each year at VCHP

The number of homeless people using the service increased year on year over the study period, perhaps due to increased awareness of the new facility amongst the population and also due to the numbers of homeless people in London increasing [4] (Fig. 1). In addition, there has also been an increase in the number of clinics that VCHP can provide. The charity opened its first clinic in 2003 and since 2007 to the present date, the charity now has six clinics situated around England (Table 3).

**Table 3:** Details of clinics run by Vision Care for Homeless People

Clinic	Location	Opening times
Crisis Skylight	Liverpool Street, London, E1	Mondays: 2.00–6.00 pm
		Wednesdays: 2.00–6.00 pm
West London Day Centre	Marylebone, London, W1	Mondays: 9.00–12.30 pm
The Broadway Centre	Goldhawk Road, London, W12	Wednesdays: 10.00–2.30 pm
Birmingham	Birmingham, B9	Mondays: 9.00–1.00 pm
Brighton	Brighton, BN1	Thursdays: 9.00–12.30 pm
Manchester	Manchester, M15	Mondays: 10.30-3.00 pm

### Visual function results

**Fig. 2:**
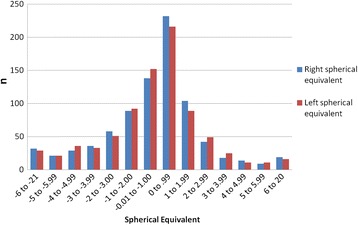
Spherical equivalent for right and left eyes for all in the sample

The spherical equivalent (SE) was calculated from prescription data available for 841 clinic users (Fig. 2). The spherical equivalent, is defined as the sphere value plus half of the cylinder value in dioptre sphere (DS) [21]. In keeping with other studies, emmetropia was defined as SE–0.50DS to +1DS, myopia as SE < −0.50DS, and hyperopia was as SE > +1DS [22].

There were no significant differences between right and left eye data when analysed using a two-sample *t*-test (t (1363) = 0.04, *p* = 0.97) and so right eye data was selected for analysis. Based on right eye data and excluding ‘not known’ data, the prevalences for emmetropia, myopia and hyperopia were 42.0 % (353/841), 37.0 % (311/841) and 21.0 % (177/841) respectively.

Figure 2 shows a normal bell shaped distribution of refractive error. The prescription issued most frequently was between Plano and +1DS [Right eye: 27.6 %, 232/841; Left eye: 25.6 %, 216/841]. The second most frequent prescription was between Plano and-1DS [Right eye: 16.4 %, 138/841; Left eye: 18.1 %, 152/841].

There were several occurrences (*n* = 51) of high refractive error of over +/−6DS; with the most hyperopic and myopic spherical equivalents prescribed being +17DS and-21DS respectively. There were a total of 841 distance prescriptions issued and 504 of these included a near addition. It was not possible to calculate the number of actual dispenses from the data available.

Unfortunately, the data on refractive error was not available for analysis for the entire sample of 1,141 patients, so it was not possible to investigate exact requirements for spectacle corrections. Using previously determined figures that an uncorrected refractive error of < = − 1DS and > = + 5DS would result in vision of approximately < =6/18 (methods) [18, 19], 34.8 % (293/841) of clinic users required spectacles.

## Discussion

This preliminary study has identified a high proportion of uncorrected refractive error in homeless people, pointing to a need for provision of regular eye examinations and refractive correction. The spectacles and lenses provided by VCHP are of good quality. The frames and ready readers are donated by Specsavers. Lenses are also donated and glazed free of charge by Essilor, Hoya, Shamir, Kent Optic, Horizon Optical and Caledonian Optical and take between 1–2 weeks to be made up. Approximately 80 % of those prescribed spectacles will collect them (personal communication).

### Gender

The sample used in this study is consistent with studies on homeless people carried out in North America in that a greater percentage of individuals were male. Our sample of homeless people seeking eye care comprised 79.1 % (*n* = 903) males, which is comparable with the North American findings of Reeve and Batty (2011) and Kleinman et al. (1996) who each reported 84 % (225/269) [23] and 70 % (254/363) [1] males in populations of homeless people in their respective samples. In the UK general population, 49.1 % are male and 50.9 % are female [24]. However, a 2010 study in Hawaii (*n* = 127) found more balanced rates of males (47 %) and females (53 %) in their sample [25] which is comparable to their population of almost equal gender (50.2 % vs’ 49.8 %) [26]. This finding might be a reflection on the small sample size but it could also perhaps suggest that generalisations between different countries and continents is not easy or necessarily valid because other factors including ethical and cultural differences and different health care infrastructures may also play a role.

### Age

The mean age for our sample shows that on average, the sample was older than for the statutory homeless. This may reflect that our clinic did not accept patients of 18 years or less, and that reading difficulties due to presbyopia (59.9 % of all prescriptions issued in our study included a reading prescription) are an incentive for older homeless people to attend for an eye examination. The Hawaii study sample had a mean age of 35 (range 7–68) [25]. Studies in Los Angeles [27] and Baltimore [28] also included those under 18. The inclusion of children and young people under 18 in these other studies limits to some extent the scope for comparison between those studies and this sample.

The charityhas a chaperone policy to enable them to see <18 year olds. The chaperone service ensures that a member of staff is present as well as a parent or guardian. To date, four Syrian refugees have been seen under this new system.

### Ethnicity

Ethnicity population figures show 87.9 % of the UK population to be classified as ‘white’ [29]. Our sample of homeless people has 80.5 % of individuals classified as ‘white’, which is comparable to rates in the normal population. ‘Asian’ people are slightly under-represented at 4 % compared to 5.9 % in the normal UK population while the percentage of ‘black’ people in our sample is 9.9 % which is comparatively high when compared to the UK population at 2.9 % [29].

There are differences, however, when judging this sample’s ethnicity against the nationality breakdown provided by National Statistics collected on statutory homeless people and rough sleepers. The data collected on statutory homeless people show 65 % ‘white’, 15 % ‘black’, and 7 % ‘Asian’ [30]. By comparison, our sample has more ‘white’ people and fewer ‘black’ and ‘Asian’ people. This may be a reflection of the EU free movement with more Europeans entering the UK. However, with such a large percentage of patients not having ethnicity details recorded (417/1,141), the data is less reliable than it could be.

### Visual function

The prevalence of myopia found within this population of homeless people was 37.0 %. This is at the high end of the range compared to rates previously found for the US population of between 16.8 % and 33.1 % [31]. The studies analysed by Pan et al. were conducted in normal populations and covered the whole population to include non-spectacle wearers [31]. This makes it difficult to compare these findings to the sample used in this study, as this sample is from a homeless population who may have been driven to attend by their need for spectacle correction. As a result, the individuals with visual problems may be more likely to attend than individuals with no obvious visual problems.

The prevalence of hypermetropia greater than +1DS found within this study was 21.0 %. A study of Americans between 40 and 80 years of age found prevalence rates of 9.95 % of hyperopia > = + 3DS [32]. Our sample had a 7.1 % (60/841) prevalence over > = + 3DS and appears comparable.

Using previously determined criteria that an uncorrected refractive error of < = − 1DS and > +5DS would result in presenting vision < = 6/18 [18, 19], 34.8 % (293/841) of the homeless people using VCHP would require a refractive correction. It is known that homeless people are a transient population who have difficulties accessing appropriate medical care [7, 9, 33] due to being unable to provide a permanent address [16, 34]. Whilst homeless people can access GP services via an “immediately necessary” route, onwards referral requires a permanent address. It is therefore not unreasonable to assume that the same issues also apply to eye care. Whilst it is possible in the UK to have free eye examinations and spectacles, the problem of providing a permanent address still arises with the GOS forms. The charity VCHP was set up in 2003 because homeless people were unwilling or unable to access mainstream services through the NHS. The cost of an eye test, if not eligble for an NHS funded eye test, and even a small cost for spectacles can prevent homeless people from being able to access refractive correction [17]. VCHP provide a comprehensive eye examination by a qualified optometrist as would be conducted in high street practice e.g. patients are dilated if clinically indicated and the Skylight Clinic used in this study also has a fundus camera available. Each clinic is attached to a homeless GP service should a referral be necessary. In addition, the charity VCHP provides spectacles free of charge and can also provide a selection of hand magnifiers (which do not require batteries) donated by the RNIB.

One difference between the VCHP clinics and the services offered on the high street is that there is limited potential for repeat visits. On the high street, it is best practice to provide a recall date. VCHP have made the decision that offering a recall date/follow up appointment perpetuates the problem by encouraging people to return to its clinics. The ideal situation is for people to be rehabilitated back into the community so they can obtain housing, jobs etc. Their future care would therefore be back in mainstream high street practice.

Our findings that 34.8 % of homeless people require a spectacle correction is consistent with the findings of Baggett et al. (2010) who report 41 % (378/966) of their sample had an unmet need for spectacles [7]. The study by Barnes et al. (2010) in Hawaii reported that two thirds of their sample was uncertain how to obtain spectacles (66.7 %) or where to access eye care (48.8 %) [25]. However, there is no information provided about how many of these actually needed a spectacle correction. It is well known that something as simple as spectacle correction can impact upon a person’s quality of life [13, 22] and that the provision of eye services and free spectacles can improve this [11, 14].

### Limitations

The study data are limited to refractive error and demographic data on the sample population. Other data, such as data on ocular pathology and eye movements, were not available via the patient files. New patient records being introduced in the clinic will facilitate more complete data collection in future studies on this population.

Despite the limitations of this study, it still remains the best data set gathered to date on the visual requirements of homeless people in the UK. Whilst other studies have attempted to study this group in other countries, some have found similarities with our data set and many of these studies have encountered similar problems. However, it is important to bear in mind that direct comparisons across the globe remain difficult due to a variety of reasons which could include climate, geography, ethical and cultural factors as well as differing social and healthcare infrastructures. Because of these global variations, it is important that baseline data regarding the characteristics of homeless people in the UK are reported so that further work can be undertaken in this vulnerable populations.

## Conclusions

This paper is the first study to investigate the visual problems and needs of homeless people in the UK and sets out a baseline of what to expect in a homeless population in the UK against which further research can be measured. Although similar work has been done in other countries such as North America, this study is unique because the UK is the only country with a National Health Service which provides free health care at the point of access.

This study has identified a high proportion of uncorrected refractive error in this sample, suggesting a need for provision of eye examinations and refractive correction for homeless people. There is scope for expansion of these clinics throughout the UK, and for further data analysis to determine the prevalence of sight threatening disease in this population and in particular to compare with a non-homeless, age-matched population.

To date, there has been no published research on the visual problems and needs of homeless people in the UK. Homelessness is likely to increase due to the current economic climate and so it is important to identify the visual needs of this vulnerable population and devise appropriate strategies to deal with them.
